# New residual feed intake criterion for longitudinal data

**DOI:** 10.1186/s12711-021-00641-2

**Published:** 2021-06-25

**Authors:** Ingrid David, Van-Hung Huynh Tran, Hélène Gilbert

**Affiliations:** GenPhySE, Université de Toulouse, INRAE, ENVT, Castanet Tolosan, France

## Abstract

**Background:**

Residual feed intake (RFI) is one measure of feed efficiency, which is usually obtained by multiple regression of feed intake (FI) on measures of production, body weight gain and tissue composition. If phenotypic regression is used, the resulting RFI is generally not genetically independent of production traits, whereas if RFI is computed using genetic regression coefficients, RFI and production traits are independent at the genetic level. The corresponding regression coefficients can be easily derived from the result of a multiple trait model that includes FI and production traits. However, this approach is difficult to apply in the case of multiple repeated measurements of FI and production traits. To overcome this difficulty, we used a structured antedependence approach to account for the longitudinality of the data with a phenotypic regression model or with different genetic and environmental regression coefficients [multi- structured antedependence model (SAD) regression model].

**Results:**

After demonstrating the properties of RFI obtained by the multi-SAD regression model, we applied the two models to FI and production traits that were recorded for 2435 French Large White pigs over a 10-week period. Heritability estimates were moderate with both models. With the multi-SAD regression model, heritability estimates were quite stable over time, ranging from 0.14 ± 0.04 to 0.16 ± 0.05, while heritability estimates showed a U-shaped profile with the phenotypic regression model (ranging from 0.19 ± 0.06 to 0.28 ± 0.06). Estimates of genetic correlations between RFI at different time points followed the same pattern for the two models but higher estimates were obtained with the phenotypic regression model. Estimates of breeding values that can be used for selection were obtained by eigen-decomposition of the genetic covariance matrix. Correlations between these estimated breeding values obtained with the two models ranged from 0.66 to 0.83.

**Conclusions:**

The multi-SAD model is preferred for the genetic analysis of longitudinal RFI because, compared to the phenotypic regression model, it provides RFI that are genetically independent of production traits at all time points. Furthermore, it can be applied even when production records are missing at certain time points.

**Supplementary Information:**

The online version contains supplementary material available at 10.1186/s12711-021-00641-2.

## Background

According to the United Nations Population Fund, the global human population is expected to reach 9.1 billion by 2050 [1]. Thus, a 70% increase in food production will be necessary to fullfil the food security requirements of this population in a context of climate change [2]. Feed efficiency in livestock production, which is defined as the ability to transform input (feed) into output (such as body weight), is a key factor in meeting this need. Different criteria to measure feed efficiency have been proposed in the literature. Feed conversion ratio (FCR), which is defined as feed intake per unit of average daily gain, is well understood by farmers and thus widely used. However, due to problems that are inherent to selection on ratio measures [3], residual feed intake (RFI) may be a preferred measure of feed efficiency [4]. RFI corresponds to the difference between an animal’s actual feed intake and that predicted on the basis of the animal’s production and maintenance requirements [4]. In growing animals, RFI is usually obtained by a multiple regression of feed intake on measures of production, body weight gain, and tissue composition [5]. On the one hand, if phenotypic regression is used for this purpose, the resulting RFI is not genetically independent of production and body weight/composition traits. Thus, to avoid detrimental effects on production traits, selection based on RFI obtained by phenotypic regression must be performed using a selection index that combines RFI and production traits with appropriate weights (derived from the covariance matrix between the components of RFI) [6, 7]. On the other hand, if genetic regression is used to derive RFI, it is then independent of the other traits at the genetic level (but not at the phenotypic level) [5]. The use of different regression coefficients for the genetic and environmental parts of the component traits (feed intake, production, body weight/composition traits), in a multiple trait approach, accounts for the multiple origins of the phenotypic correlations [8, 9] and provides a RFI that is independent of the other traits at the genetic level.

Using automatic self-feeders [10–14], individual feed intake can be recorded whenever an animal accesses its feeder. Since repeated recordings of body weight and body composition are also possible, it is conceivable to compute repeated measurements of RFI at various ages [15]. Compared to the use of an average RFI over the growing period, the main advantage of such longitudinal RFI data is the ability to describe changes in the trait over time and to provide more accurate estimates of breeding values for genetic selection [16]. However, applying a multiple trait model to account for covariances between measurements at different time points becomes problematic when there are more than five time points. Several flexible mixed-model approaches that require estimation of fewer parameters than the multiple trait model have been proposed for the analysis of longitudinal data in the genetic context: random regression (RR), character process (CP), and structured antedependence (SAD) models [17–19]. To evaluate longitudinal RFI and propose appropriate selection criteria, extension of these longitudinal models to the multivariate case is necessary; indeed, a multiple trait analysis of longitudinal FI and production traits would allow the genetic and environmental covariances between FI and production traits to be estimated. Such an extension is not straightforward for the CP model [20] and may result in convergence issues for the RR model due to the large number of parameters to be estimated ($\sum \nolimits_{i = 1}^{n} \frac{{d_{i} \left( {d_{i} + 1} \right)}}{2}$ per matrix of random effects for $n$ traits for a polynomial of degree $d_{i}$ for trait $i$).

The objective of our study was to propose a multivariate SAD model to estimate the genetic and environmental covariances between FI and production traits. Based on these estimates, we propose the use of genetic regression coefficients for the genetic and environmental parts of the component traits of RFI to obtain genetic and environmental components of longitudinal RFI that will be independent of production traits at the genetic level. The proposed multivariate SAD regression model was applied to pig data and compared to a longitudinal model based on phenotypic regression at each time point.

## Methods

### SAD model for longitudinal RFI

To present the general form of the SAD model used in this study, RFI is considered as a function of four component traits, as usually applied in pigs [7, 21]: feed intake (FI), average daily gain (ADG), metabolic body weight (MBW), and backfat thickness (BF). The extension to include different components or additional components is straightforward. For the sake of simplicity, these ‘production and maintenance traits’ are hereafter referred to as ‘production traits’.

Let $ADG_{ij}$, $MBW_{ij}$, $BF_{ij}$, and $FI_{ij}$ be the observed values for ADG, MBW, BF, and FI of animal $i$ at time $t_{j}$ ($j$ = 1 to $n$), and ${\mathbf{ADG}}_{j}$, ${\mathbf{MBW}}_{j}$, ${\mathbf{BF}}_{j}$, and ${\mathbf{FI}}_{j}$ the corresponding vectors of observations across animals for time point $j$. Here, we propose to model longitudinal RFI in two ways: longitudinal RFI obtained by a phenotypic regression of FI on the production traits at each time point (phenotypic regression model), which is the most commonly used and easiest way to compute RFI [22], versus RFI obtained with a genetic and environmental regression on the production traits, which corresponds to a multi-SAD genetic regression to model longitudinal RFI (multi-SAD regression model). In both cases, we used the SAD approach to account for covariances between measurements at different time points [23], and to account for covariances between FI and production traits in the multi-SAD regression model. Like the RR model, the SAD approach attempts to model the form of the random effects function to provide the most appropriate and parsimonious covariance function for the different random effects in the model. To do so, the RR approach uses (orthogonal) polynomials of time. Then, the covariance matrix between time points is derived from the covariance matrix of the random coefficients. Extension to the multiple trait case is straightforward and is done by considering the covariance matrix of the random coefficients for each trait involved, i.e. the random coefficients for different traits are correlated. In the single trait case, the SAD approach models the random effects function by assuming that a random effect at time point $t_{j}$ can be explained by its previous values (i.e. at time point $t_{k}$, $k < j$). A random effect at time point $t_{j}$ is then obtained by a regression on its preceding value(s). The covariance matrix between time points can be derived from the value of the regression coefficients and the variance of the error term in the regression. Extension to the multiple-trait situation is obtained by assuming that, in addition to the within-trait relationship, a random effect of one trait can be a function of the same random effect of the other traits considered, i.e. by adding the values of that random effect for the other traits in the regression. The covariance matrix between time points and traits is then derived from the values of the regression coefficients and the variances of the error terms. To reduce the number of parameters to be estimated, in the SAD approach it is generally assumed that the regression coefficients (called antedependence and cross-antedependence parameters) and the variance of the error term (called innovation variance) are functions of time [17, 24].

#### Phenotypic regression model

The equation to obtain the genetic and environmental components of RFI for animal $i$ at time point $t_{j}$ is:

$$FI_{ij} = \mu_{FI,ij} + b_{ADGj} ADG_{ij} + b_{MBWj} MBW_{ij} + b_{BFj} BF_{ij} + u_{RFI,ij} + e_{RFI,ij} ,$$

where $\mu_{FI,ij}$ represents the fixed effects at time point $t_{j}$; $b_{ADGj}$, $b_{MBWj}$, and $b_{BFj}$ are the phenotypic regression coefficients of FI on ADG, MBW, and BF for time point $t_{j}$ $\left( {b_{ADGj} = \frac{{cov\left( {{\mathbf{ADG}}_{{\text{j}}} ,{\mathbf{FI}}_{{\text{j}}} } \right)}}{{\sigma_{ADGj}^{2} }} ,\;b_{MBWj} = \frac{{cov\left( {{\mathbf{MBW}}_{{\text{j}}} ,{\mathbf{FI}}_{{\text{j}}} } \right)}}{{\sigma_{MBWj}^{2} }},\;{\text{and,}}\;b_{BFj} = \frac{{cov\left( {{\mathbf{BF}}_{{\text{j}}} ,{\mathbf{FI}}_{{\text{j}}} } \right)}}{{\sigma_{BFj}^{2} }}} \right)$, $u_{RFI,ij}$ is the additive genetic effect for RFI of animal $i$ at time point $t_{j}$, and $e_{RFI,ij}$ is the random residual. Genetic and residual effects of different time points are not independent. To account for covariances between time points, the SAD approach models a random effect at time point $t_{j}$ by a regression on the preceding observations, leading to:

$$\begin{aligned} u_{RFI,ij} &= \theta_{uRFI,j} u_{{RFI,i\left( {j - 1} \right)}} + \varepsilon_{uRFI,ij} \hfill \\ e_{RFI,ij} &= \theta_{eRFI,j} e_{{RFI,i\left( {j - 1} \right)}} + \varepsilon_{eRFI,ij} \end{aligned},$$

where $\theta_{uRFI,j}$ and $\theta_{eRFI,j}$ are the antedependence parameters at time point $t_{j}$ for the genetic and residual components of RFI, and $\varepsilon_{uRFI,ij}$ and $\varepsilon_{eRFI,ij}$ are the genetic and residual error terms at time point $t_{j}$, which are assumed to be random normally distributed effects with a mean 0 and innovation variances ${\mathbf{A}}\sigma_{\varepsilon uRFI,j}^{2}$ and ${\mathbf{I}}\sigma_{\varepsilon eRFI,j}^{2}$, respectively, where ${\mathbf{A}}$ is the numerator genetic relationship matrix and ${\mathbf{I}}$ an identity matrix of appropriate size. To reduce the number of parameters to be estimated, antedependence parameters and innovation variances are considered as functions of time: $\theta_{s,j} = \sum \nolimits_{q = 0}^{{\beta_{s} }} a_{sq} t_{j}^{q}$ for a function of degree $\beta_{s}$ ($s = uRFI$ or $eRFI$), and $\sigma_{\varepsilon s,j}^{2} = exp\left( { \sum \nolimits_{q = 0}^{{\gamma_{s} }} d_{sq} t_{j}^{q} } \right)$ for a function of degree $\gamma_{s}$ [25]. Hereafter, the notation SAD $\beta_{s} \gamma_{s}$ is used to shorten the description of the SAD model. Then, the variance–covariance matrices ${\mathbf{G}}$ and ${\mathbf{P}}$ of the genetic and environmental components of RFI can be obtained by calculating their inverse using a Cholesky decomposition [26]: ${\mathbf{P}}^{ - 1} = {\mathbf{L^{\prime}D}}^{ - 1} {\mathbf{L}}$, where ${\mathbf{D}}$ is a diagonal matrix with innovation variances for RFI as components, and ${\mathbf{L}}$ is a lower triangular matrix with 1s on the diagonal and the negatives of the antedependence parameters for RFI as off-diagonal entries (the same reasoning applies to the ${\mathbf{G}}\user2{ }$ matrix). The phenotypic value of RFI for animal $i$ at time point $t_{j}$ in the phenotypic regression model is then computed as $RFI_{ij} = u_{RFI,ij} + e_{RFI,ij}$. In this model, the genetic covariance matrix between RFI and longitudinal production traits is not estimated which prevents the computation of an optimum selection index of RFI and production traits.

#### Multi-SAD regression model

The multi-SAD regression model is derived from a multiple trait SAD model [24, 27]. A detailed description of the general form of the multiple trait SAD model is given in David et al. [24]. The set of model equations is as follows:

$$\left\{ \begin{aligned} ADG_{ij} & = \mu_{ADG,ij} + u_{ADG,ij} + e_{ADG,ij} \\ MBW_{ij} & = \mu_{MBW,ij} + u_{MBW,ij} + e_{MBW,ij} \\ BF_{ij} & = \mu_{BF,ij} + u_{BF,ij} + e_{BF,ij} \\ FI_{ij} & = \mu_{FI,ij} + u_{FI,ij} + e_{FI,ij} \\ \end{aligned} \right.,$$

where $\mu_{ADG,ij}$, $\mu_{MBW,ij}$, $\mu_{BF,ij}$, and $\mu_{FI,ij}$ represent the fixed effects at time point $t_{j}$, $u_{ADG,ij}$, $u_{MBW,ij}$, $u_{BF,ij}$, and $u_{FI,ij}$ are the additive genetic random effect functions, and $e_{ADG,ij}$, $e_{MBW,ij}$, $e_{BF,ij}$, and $e_{FI,ij}$ are the random residual functions for ADG, MBW, BF, and FI, respectively. To account for covariances between traits and between time points within a trait, the SAD approach makes it possible to model the form of the random effects function with a regression on the preceding observations and on correlated traits. To model RFI, the following genetic random effects functions are used:

1 $$\begin{aligned} u_{ADG,ij} & = \theta_{uADG,j} u_{{ADG,i\left( {j - 1} \right)}} + \varepsilon_{uADG,ij} \\ u_{MBW,ij} & = \theta_{uMBW,j} u_{{MBW,i\left( {j - 1} \right)}} + \varepsilon_{uMBW,ij} \\ u_{BF,ij} & = \theta_{uBF,j} u_{{BF,i\left( {j - 1} \right)}} + \varepsilon_{uBF,ij} \\ u_{FI,ij} & = \theta_{uFI,j} u_{{FI,i\left( {j - 1} \right)}} + b_{u,ADGj} u_{ADG,ij} + b_{u,MBWj} u_{MBW,ij} + b_{u,BFj} u_{BF,ij} + \varepsilon_{uFI,ij} \\ \end{aligned} ,$$

where $\theta_{uADG,j}$, $\theta_{uMBW,j}$, $\theta_{uBF,j}$, and $\theta_{uFI,j}$ are the antedependence parameters at time point $t_{j}$ for the genetic components of ADG, MBW, BF, and FI, respectively; $b_{u,ADGj}$, $b_{u,MBWj}$, and $b_{u,BFj}$ are the genetic regression coefficients (cross-antedependence parameters) of FI on ADG, MBW, and BF at time point $t_{j}$ $\left( {b_{u,ADGj} = \frac{{cov\left( {{\mathbf{u}}_{{{\text{ADG}},{\text{j}}}} ,{\mathbf{u}}_{{{\text{FI}},{\text{j}}}} } \right)}}{{\sigma_{uADG,j}^{2} }} ,\;b_{u,MBWj} = \frac{{cov\left( {{\mathbf{u}}_{{{\text{MBW}},{\text{j}}}} ,{\mathbf{u}}_{{{\text{FI}},{\text{j}}}} } \right)}}{{\sigma_{uMBW,j}^{2} }},\;{\text{and}}\;b_{u,BFj} = \frac{{cov\left( {{\mathbf{u}}_{{{\text{BF}},{\text{j}}}} ,{\mathbf{u}}_{{{\text{FI}},{\text{j}}}} } \right)}}{{\sigma_{uBF,j}^{2} }}} \right)$ and ${{\varvec{\upvarepsilon}}}_{{{\text{uADG}},{\text{j}}}}$, ${{\varvec{\upvarepsilon}}}_{{{\text{uMBW}},{\text{j}}}}$, ${{\varvec{\upvarepsilon}}}_{{{\text{uBF}},{\text{j}}}}$, and ${{\varvec{\upvarepsilon}}}_{{{\text{uFI}},{\text{j}}}}$ are random normally distributed effects (error terms) with mean 0 and innovation variances ${\mathbf{A}}\sigma_{\varepsilon uADG,j}^{2}$, ${\mathbf{A}}\sigma_{\varepsilon uMBW,j}^{2}$, ${\mathbf{A}}\sigma_{\varepsilon uBF,j}^{2}$, and ${\mathbf{A}}\sigma_{\varepsilon uFI,j}^{2}$, respectively. The same approach is used to model the residual random function:

2 $$\begin{aligned} e_{ADG,ij} & = \theta_{eADG,j} e_{{ADG,i\left( {j - 1} \right)}} + \varepsilon_{eADG,ij} \\ e_{MBW,ij} & = \theta_{eMBW,j} e_{{MBW,i\left( {j - 1} \right)}} + \varepsilon_{eMBW,ij} \\ e_{BF,ij} & = \theta_{eBF,j} e_{{BF,i\left( {j - 1} \right)}} + \varepsilon_{eBF,ij} \\ e_{FI,ij} & = \theta_{eFI,j} e_{{FI,i\left( {j - 1} \right)}} + b_{e,ADGj} e_{ADG,ij} + b_{e,MBWj} e_{MBW,ij} + b_{e,BFj} e_{BF,ij} + \varepsilon_{eFI,ij} \\ \end{aligned},$$

where $\theta_{eADG,j}$, $\theta_{eMBW,j}$, $\theta_{eBF,j}$, and $\theta_{eFI,j}$ are the antedependence parameters at time point $t_{j}$ for the residual components of ADG, MBW, BF, and FI, respectively; $b_{eADGj}$, $b_{eMBWj}$, and $b_{eBFj}$ are the environmental regression coefficients of FI on ADG, MBW, and BF at time point $t_{j}$, and ${{\varvec{\upvarepsilon}}}_{{{\text{eADG}},{\text{j}}}}$, ${{\varvec{\upvarepsilon}}}_{{{\text{eMBW}},{\text{j}}}}$, ${{\varvec{\upvarepsilon}}}_{{{\text{eBF}},{\text{j}}}}$, and ${{\varvec{\upvarepsilon}}}_{{{\text{eFI}},{\text{j}}}}$ are random normally distributed effects (error terms) with mean 0 and innovation variance ${\mathbf{I}}\sigma_{\varepsilon eADG,j}^{2}$, ${\mathbf{I}}\sigma_{\varepsilon eMBW,j}^{2}$, ${\mathbf{I}}\sigma_{\varepsilon eBF,j}^{2}$, and ${\mathbf{I}}\sigma_{\varepsilon eFI,j}^{2} .$

As in the phenotypic regression model, antedependence parameters and innovation variances are assumed to be continuous functions of time to reduce the number of parameters to be estimated (i.e. $\theta_{s,j} = \sum \nolimits_{q = 0}^{{\beta_{s} }} a_{sq} t_{j}^{q}$ for a function of degree $\beta_{s}$ ($s \in \left\{ {eADG, uADG, eMBW, uMBW, eBF, uBF,eFI, } \right. \left. {uFI} \right\}$) and $\sigma_{s,j}^{2} = exp\left( { \sum \nolimits_{q = 0}^{{\gamma_{s} }} d_{sq} t_{j}^{q} } \right)$ for a function of degree $\gamma_{s}$. Regression coefficients (cross-antedependence parameters) are also assumed to be functions of time: $b_{s,j} = \sum \nolimits_{q = 0}^{{\delta_{s} }} c_{sq} t_{j}^{q}$ for a function of degree $\delta_{s}$ ($s \in \left\{ {eADG, uADG, eMBW, uMBW, eBF, uBF} \right\}$). Given Eqs. (1) and (2), it is possible to obtain the genetic and residual components of RFI*, computed with the multi-SAD regression model, by adjusting FI for ADG, MBW and BF at the genetic and environmental levels. Then, we obtain RFI*, which is independent from ADG, MBW and BF at the genetic level as:

$$\begin{aligned} u_{RFI,ij}^{*} &= u_{FI,ij} + \theta_{uFI,j} \left( {u^{*}_{{RFI,i\left( {j - 1} \right)}} - u_{{FI,i\left( {j - 1} \right)}} } \right) - b_{u,ADGj} u_{ADG,ij} - b_{u,MBWj} u_{MBW,ij} - b_{u,BFj} u_{BF,ij} \\ e_{RFI,ij}^{*} &= e_{FI,ij} + \theta_{eFI,j} \left( {e^{*}_{{RFI,i\left( {j - 1} \right)}} - e_{{FI,i\left( {j - 1} \right)}} } \right) - b_{u,ADGj} e_{ADG,ij} - b_{u,MBWj} e_{MBW,ij} - b_{u,BFj} e_{BF,ij} \end{aligned}.$$

The phenotypic value for RFI for animal $i$ at time point $t_{j}$ in the multi-SAD model is $RFI_{ij}^{*} = u_{RFI,ij}^{*} + e_{RFI,ij}^{*}$. See Additional file 1 for a demonstrative example.

The variance–covariance matrices ${\mathbf{G}}^{*}$ of the genetic component of RFI* can be obtained by calculating its inverse using the following Cholesky decomposition: ${\mathbf{G}}^{* - 1} = {\mathbf{L}}^{{*^{\prime}}} {\mathbf{D}}^{* - 1} {\mathbf{L}}^{*}$, where ${\mathbf{D}}^{*}$ is a diagonal matrix with genetic innovation variances for FI as components, and ${\mathbf{L}}^{*}$ is a lower triangular matrix with 1s on the diagonal and the negatives of the genetic antedependence parameters for FI as off-diagonal entries [cross antedependence is excluded, (see Additional file 1 for the demonstration)]. The environmental covariance matrix ${\mathbf{P}}^{*}$ of RFI* and production traits is obtained using the following covariance function: ${\mathbf{P}}^{*} = {\mathbf{BP}}_{{\mathbf{T}}} {\mathbf{B^{\prime}}}$, where ${\mathbf{P}}_{{\mathbf{T}}}$ is the environmental covariance matrix for FI and production traits, and ${\mathbf{B}}$ is a lower triangular matrix of regression coefficients:

$${\mathbf{B}} = \left[ {\begin{array}{*{20}c} {{\mathbf{I}}_{ADG} } & & & \\ \mathbf{0} & {{\mathbf{I}}_{MBW} } & & \\ \mathbf{0} & \mathbf{0} & {{\mathbf{I}}_{BF} } & \\ {{\mathbf{b}}_{eADF/FI} } & {{\mathbf{b}}_{eMBW/FI} } & {{\mathbf{b}}_{eBF/FI} } &{{\mathbf{I}}_{FI} } \\ \end{array} } \right],$$

where ${\mathbf{b}}_{es/FI}$ $( s \in \left\{ {ADG,MBW, BF\} } \right.$) are lower triangular matrices with the negative of the genetic cross-antedependence parameters (genetic regression coefficients) on the diagonal and the negatives of the product of environmental antedependence parameters with genetic cross-antedependence parameters as off-diagonal entries $\left( {{\text{for}}\; {\text{cell}} \left( {i,j} \right),i < j: - \prod \nolimits_{k = 2}^{j} \theta_{eFI,k} b_{u,si} } \right)$:

$${\mathbf{b}}_{s/FI} = \left[ {\begin{array}{*{20}c} { - b_{u,s1} } & & & & \\ { - \theta_{eFI,2} b_{u,s1} } & \cdots & & & \\ { - \prod \limits_{k = 2}^{j} \theta_{eFI,k} b_{u,s1} } & \cdots & { - b_{u,sj} } & & \\ \vdots & \vdots & \vdots & \cdots & {} \\ \cdots & \cdots & { - \prod \limits_{k = 2}^{n} \theta_{eFI,k} b_{u,sj} } & \cdots & { - b_{u,sn} } \\ \end{array} } \right],$$

for $n$ measurement times. By construction, RFI* at time point $t_{j}$ is then independent of ADG, MBW, and BF at all time points at the genetic level.

#### Model implementation

The phenotypic regression and multi-SAD regression models can be implemented using the ASReml software [28] and the OWN Fortran program developed by David, which are freely available on the Zenodo platform [29]. The degrees of the polynomials of time for each function can be selected by comparing nested models using likelihood ratio tests. In the multi-SAD regression model, to reduce the number of models to be compared, we selected the specification of the antedependence and innovation variances for each trait independently, and then the cross-antedependence specification. For the first part of the selection process, we started with the most parsimonious model (smallest degree for the polynomial functions of time), and then increasing the degree of the polynomial functions of time (alternating the antedependence parameter and the innovation variance) until there is no further significant improvement of the model. Next, an increase in the degree of the cross-antedependence parameters is tested. For this step, we increased the degree of all cross antedependence parameters together. In addition, to reduce computing time and avoid convergence issues, antedependence and innovation variance parameters for ADG, MBW, and BF were considered as known (i.e. fixed to their values estimated in the first step of the selection process).

Both models provide estimated breeding values (EBV) for each time point $t_{j}$ of observation (TEBV: Time EBV). No simple procedure exists to use ‘raw’ TEBV for selection, as it requires the $n$ TEBV to be accounted for. Instead, animals should be selected based on the trajectory of their TEBV over time. This selection requires a limited number of descriptive parameters of these trajectories, which are usually obtained by eigendecomposition of the genetic covariance matrix [15, 30]. It has been shown that the two first eigenvectors obtained with longitudinal models are usually sufficient to describe the trajectory of feed efficiency [15]. The corresponding summarised estimated breeding values (SBV1 and SBV2), which are the product of the vector of TEBV with the first or the second eigenvectors, generally correspond to the EBV for average feed efficiency and the EBV for the main slope of the trajectory over time, respectively [15].

### Application to pig data

We used data from 2435 French Large White boars, castrated males, and gilts from nine generations of two lines that were divergently selected for RFI (called hereafter high RFI line and low RFI line). All pigs were raised after weaning on the Rouillé experimental farm (Vienne, France, 10.15454/1.5572415481185847E12) [21]. The animals were selected based on their RFI over an 18-week test period, starting at ~ 10 weeks of age, which was obtained by a defined phenotypic regression of FI on ADG, MBW, and BF over the test period (i.e. one observation per animal). From each farrowing batch, 48 pigs from at least six litters were moved to a growing-finishing room at ~ 10 weeks of age, with four groups of 12 animals (boars separated from females and castrated males) placed in pens that were each equipped with a single-place electronic feeder ACEMA 64 (Pontivy, France; [31]). Over the 18-week test period, the animals were fed a pelleted diet of cereals and soybean meal with 10 MJ NE/kg and 160 g CP/kg, and a minimum of 0.80 g digestible Lys/MJ NE. The animals had free access to water. Feed intake was recorded each time a pig accessed the feeder. For 16 consecutive weeks of the test period (from 11 to 26 weeks of age), body weight (BW) was recorded weekly for the males, while females and castrated males were weighed at 11, 15, 19, and 23 weeks of age, and more frequently if the test lasted for more than 23 weeks. Backfat thickness was measured ultrasonically on males at around 35, 65, 90, and 95 kg body weight, as the average of six measurements that were recorded at three areas of the body: the neck, the back, and the kidney, on both sides of the spine. For the females and castrated males, BF was measured in the same way, but at 11, 15, 19, and 23 weeks of age.

For longitudinal analyses, phenotypes were computed at weekly intervals. Average daily gain of animal $i$ at week $j$ was calculated over a 4-week period following [32] as $ADG_{ij} = \frac{{BW_{{i\left( {j + 2} \right)}} - BW_{{i\left( {j - 2} \right)}} }}{{age_{{i\left( {j + 2} \right)}} - age_{{i\left( {j - 2} \right)}} }}$ and MBW was calculated as $MBW_{ij} = BW_{ij}^{0.6}$. $BF_{ij}$ corresponded to the measurements of BF on animal $i$ at week $j$. Outliers, as defined in Huynh-Tran et al. [15], were removed from the analysis. Data for the first week in the growing-finishing room (11 weeks of age), which was considered to be an adaptation period for the animals, and for weeks corresponding to 25 and 26 weeks of age, as the number of pigs weighed per week decreased at the end of the test period (animals already slaughtered), were also removed from the dataset. The final data set included production and FI records over a 10-week period from ~ 13 to ~ 22 weeks of age, which will be denoted as weeks 1 to 10, hereafter. The relationship matrix for use in the analyses was built from pedigree, which included 3986 animals.

### Comparison of models

Given the poor properties for RFI obtained from the phenotypic regression model (genetically correlated with production traits), our aim was to compare RFI obtained with the phenotypic regression and RFI obtained with the multi-SAD regression models, in order to evaluate the advantages of applying the multi-SAD model. The core issue was to evaluate whether the EBV for RFI obtained with the multi-SAD model are sufficiently different from those obtained with the phenotypic regression model to consider applying such a complex model to the data. Fixed effects included in the two models were the same for all traits: week (10 levels), batch (66 levels), sex (3 levels), birth herd (2 levels), age at the start of the test (covariate), and pen (16 levels). To apply the phenotypic regression model, missing phenotypes for BW and BF were replaced by their predictions from linear interpolations then MBW and ADG for all weeks were computed using the predicted BW, as described in Huynh-Tran et al. [32]. To make the phenotypic and multi-SAD regression models comparable, these phenotypes were also used in the multi-SAD regression model, although this model can cope with missing values. Spearman rank correlations were used to compare the weekly EBV and the first and second SBV obtained with the two models. In addition, the trajectories of individual EBV over weeks were classified into groups by a non-hierarchical k-mean analysis [33] for both models. Finally, we evaluated the effect of the divergent selection on RFI obtained by a phenotypic regression at the test level (i.e. one observation over the test period) on the profile of RFI from the multi-SAD regression model over weeks by comparing changes in SBV due to selection.

## Results

The descriptive statistics of the traits per week are in Table 1. The phenotype of all traits increased with time. The coefficients of variation were in the same range for ADG, MBW and FI (ranging from 0.13 to 0.21) and lower for MBW (ranging from 0.07 to 0.10). The SAD models that were retained for the phenotypic regression model and the multi-SAD regression model are described in Table 2. The degrees of the functions of the cross-antedependence parameters (genetic and environmental regression coefficients of FI on ADG, MBW, and BF) were all equal to 1 in the multi-SAD regression model. The corresponding equations are in Additional file 2. The estimates of heritabilities of FI and production traits with the multi-SAD model are shown in Additional file 3: Figure S1. All heritabilities increased with time from 0.20 to 0.39 for FI, from 0.32 to 0.39 for ADG, from 0.21 to 0.60 for MBW, and from 0.27 to 0.48 for BF. Estimates of the genetic and phenotypic correlations between weeks for FI and production traits that were obtained with the multi-SAD model are shown in Additional file 4: Figure S2. Similar patterns were observed for FI and production traits, i.e. within trait, estimates of the genetic and phenotypic correlations decreased as the time between measurements increased, and estimates of the phenotypic correlations were slightly weaker than estimates of the genetic correlations. Within week, estimates of the genetic correlation between FI and ADG ranged from 0.60 and 0.75. They were lower between FI and MBW (ranging from 0.15 to 0.37), and between FI and BF (ranging from 0.10 to 0.28). Estimates of the phenotypic correlations followed the same patterns but with lower values.

**Table 1:** Descriptive statistics of the data

Week	ADG (10× g/d)		MBW (10× kg^0.6^)		BF (0.1× mm)		FI (10× g/d)	
	Mean ± sd	% missing	Mean ± sd	% missing	Mean ± sd	% missing	Mean ± sd	% missing
1	74 ± 14	12	90 ± 9	52	75 ± 10	87	168 ± 36	< 1
2	77 ± 14	59	96 ± 10	59	76 ± 13	95	181 ± 38	< 1
3	77 ± 13	55	106 ± 10	8	107 ± 20	46	193 ± 39	< 1
4	81 ± 14	61	112 ± 10	58	107 ± 16	92	202 ± 40	< 1
5	84 ± 15	8	118 ± 10	53	104 ± 14	89	213 ± 41	< 1
6	86 ± 14	60	124 ± 11	52	103 ± 16	87	221 ± 42	< 1
7	89 ± 15	53	133 ± 10	< 1	128 ± 27	37	229 ± 43	< 1
8	91 ± 16	55	138 ± 10	13	122 ± 23	86	234 ± 44	< 1
9	81 ± 17	14	145 ± 10	32	126 ± 19	82	242 ± 44	< 1
10	87 ± 18	74	150 ± 10	35	123 ± 18	77	245 ± 44	6

*ADG* average daily gain, *MBW* metabolic body weight, *BF* backfat thickness, *FI* feed intake

**Table 2:** SAD models retained for the genetic and environmental components in the phenotypic regression and multi-SAD regression models

	Genetic component	Environmental component
Multi-SAD regression model		
ADG	SAD00	SAD00
MBW	SAD00	SAD01
BF	SAD00	SAD01
FI	SAD11^a^	SAD12^a^
Phenotypic regression model		
RFI	SAD11	SAD12

SAD $\alpha \beta$ implies a polynomial function of degree $\alpha$ for the antedependence parameter and of degree $\beta$ for the innovation variance

*ADG* average daily gain, *MBW* metabolic body weight, *BF* backfat thickness, *FI* feed intake, *RFI* residual feed intake

^a^In addition, the degree of the functions of the cross-antedependence parameters in the model for FI were all equal to 1 for the genetic and residual parts

Estimates of the regression coefficients obtained with the phenotypic regression and multi-SAD regression models are in Table 3. Estimates of the phenotypic regression coefficient for ADG were quite stable from week 1 to 5, ranging from 0.87 to 1.00, then increased to 1.32 in week 6, and finally decreased linearly to a value of 1.00 in week 10. Estimates of the phenotypic regression coefficient for MBW increased from week 1 to 3 (from 1.04 to 1.29), then tended to decrease until week 9 (0.90), and increased again in week 10 (1.17). Estimates of the phenotypic regression coefficient for BF were quite stable over time, ranging from 0.34 to 0.47. Given the form of the multi-SAD regression model used (Table 2), estimates of all the genetic and environmental regression coefficients changed linearly with time. Estimates of the genetic regression coefficient for ADG decreased with time from 1.13 to 0.56 (slope = − 0.06 ± 0.02), while estimates of the environmental regression coefficient were quite stable (ranging from 0.92 to 0.93, with a slope that was not significantly different from 0); for MBW, estimates of both the genetic and environmental regression coefficients decreased with time from 1.48 to − 0.08 (slope = − 0.17 ± 0.03) and from 0.48 to 0.15 (slope = − 0.04 ± 0.01), respectively. Finally, for BF, estimates of the genetic regression coefficients remained stable over time at 0.18, while estimates of the environmental regression coefficients ranged from 0.22 to 0.28 (slope = 0.006 ± 0.008).

**Table 3:** Estimates of phenotypic, genetic, and environmental regression coefficients (± se) for each week and production trait with the phenotypic regression ($b_{ADG} , b_{MBW} , b_{BF}$) and multi-SAD regression models ($b_{u,ADG} , b_{u,MBW} , b_{u,BF} , b_{e,ADG} , b_{e,MBW} , b_{e,BF}$)

Week	$b_{ADG}$	$b_{u,ADG}$	$b_{e,ADG}$	$b_{MBW}$	$b_{u,MBW}$	$b_{e,MBW}$	$b_{BF}$	$b_{u,BF}$	$b_{e,BF}$
1	0.89 ± 0.05	1.13 ± 0.10	0.92 ± 0.04	1.04 ± 0.09	1.48 ± 0.15	0.48 ± 0.07	0.38 ± 0.05	0.18 ± 0.08	0.22 ± 0.04
2	0.90 ± 0.05	1.07 ± 0.08	0.92 ± 0.04	1.24 ± 0.09	1.30 ± 0.13	0.44 ± 0.06	0.47 ± 0.05	0.18 ± 0.07	0.22 ± 0.04
3	0.87 ± 0.05	1.00 ± 0.07	0.92 ± 0.03	1.29 ± 0.08	1.13 ± 0.11	0.41 ± 0.05	0.40 ± 0.04	0.18 ± 0.04	0.23 ± 0.03
4	0.97 ± 0.04	0.94 ± 0.06	0.92 ± 0.03	1.21 ± 0.08	0.96 ± 0.09	0.40 ± 0.04	0.35 ± 0.04	0.18 ± 0.04	0.24 ± 0.03
5	1.00 ± 0.04	0.88 ± 0.05	0.92 ± 0.02	1.25 ± 0.07	0.79 ± 0.08	0.33 ± 0.04	0.35 ± 0.04	0.18 ± 0.04	0.24 ± 0.02
6	1.32 ± 0.04	0.82 ± 0.05	0.93 ± 0.02	1.10 ± 0.07	0.62 ± 0.08	0.30 ± 0.04	0.34 ± 0.03	0.18 ± 0.04	0.25 ± 0.02
7	1.23 ± 0.04	0.75 ± 0.06	0.93 ± 0.03	1.19 ± 0.07	0.44 ± 0.09	0.26 ± 0.04	0.34 ± 0.03	0.18 ± 0.04	0.26 ± 0.03
8	1.15 ± 0.04	0.69 ± 0.07	0.93 ± 0.03	1.11 ± 0.08	0.27 ± 0.11	0.22 ± 0.05	0.42 ± 0.03	0.18 ± 0.05	0.26 ± 0.03
9	1.06 ± 0.04	0.63 ± 0.08	0.93 ± 0.04	0.90 ± 0.08	0.10 ± 0.13	0.19 ± 0.06	0.42 ± 0.03	0.18 ± 0.06	0.27 ± 0.04
10	1.00 ± 0.04	0.56 ± 0.10	0.93 ± 0.04	1.17 ± 0.08	− 0.08 ± 0.15	0.15 ± 0.07	0.34 ± 0.03	0.18 ± 0.08	0.28 ± 0.04

*ADG* average daily gain, *MBW* metabolic body weight, *BF* backfat thickness, *FI* feed intake

**Fig. 1:**
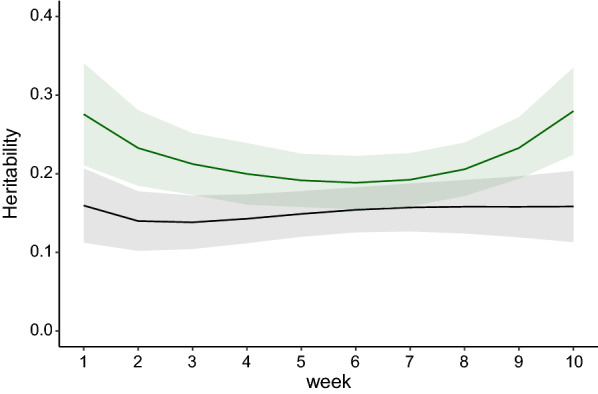
Heritability estimates of RFI over a period of 10 weeks obtained with the phenotypic regression (green) and with the multi-SAD regression (black) models. Shaded area = 95% confidence interval

Heritability estimates for RFI over time obtained with the phenotypic regression and the multi-SAD regression models were moderately high (see Fig. 1). Those estimated with the multi-SAD regression model remained quite stable over time, ranging from 0.14 ± 0.04 to 0.16 ± 0.05, and those estimated with the phenotypic regression model were slightly higher and showed a U-shaped profile, with a minimum value (0.19 ± 0.06) in week 6 and a maximum value in week 10 (0.28 ± 0.06). Based on 95% confidence intervals, only the heritability estimates obtained for weeks 1 to 3 and week 10 differed significantly between the two models.

**Fig. 2:**
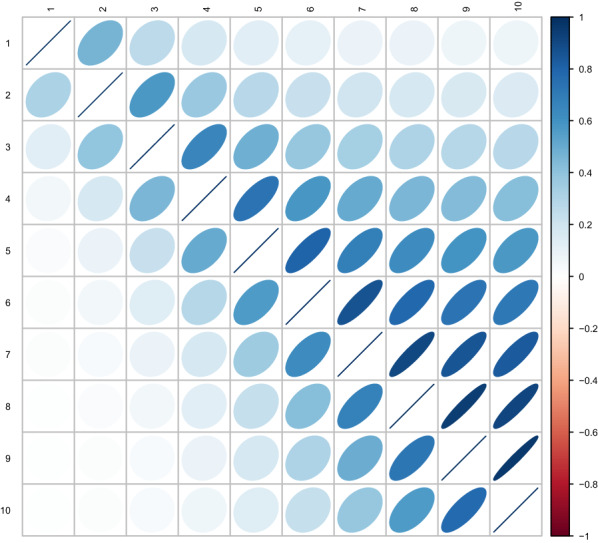
Estimates of genetic correlations between RFI in different weeks obtained with the phenotypic regression model (above the diagonal) and multi-SAD regression model (below the diagonal)

The genetic correlations between RFI at different time points estimated by the phenotypic and the multi-SAD regression models followed the same pattern over time between the two models (Fig. 2), but those obtained with the phenotypic regression model were higher, i.e. (1) estimates of genetic correlations between two successive time points increased over time and ranged from 0.46 to 0.97 with the phenotypic regression model, and from 0.32 to 0.77 with the multi-SAD regression model; and (2) genetic correlations tended to decrease as time between measurements increased, with estimates of 0.10 and 0.00 between the first and last week of estimation with the phenotypic regression and multi-SAD regression model, respectively.

**Fig. 3:**
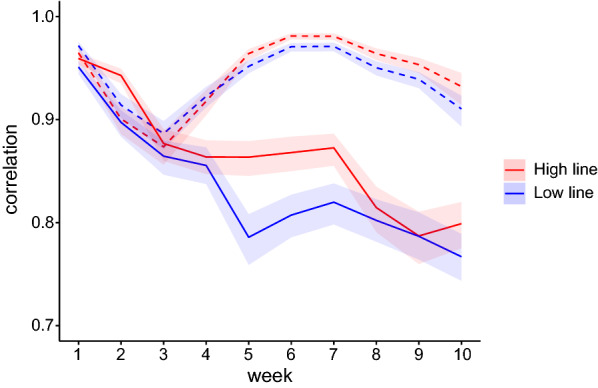
Spearman correlations per week and per line between phenotypes^a^ (dashed lines) and estimated breeding values^b^ (solid lines) for RFI obtained with the phenotypic regression and the multi-SAD regression models. ^a^For week *j*, $\rho \left( {\widehat{RFI}_{.j} ,\widehat{RFI}_{.j}^{*} } \right)$ where phenotypes of animal $i$ at week $j$ are $\widehat{RFI}_{ij} = \hat{u}_{RFI,ij} + \hat{e}_{RFI,ij}$ and $\widehat{RFI}_{ij}^{*}$ = $\hat{u}_{RFI,ij}^{*} + \hat{e}_{RFI,ij}^{*}$ for the phenotypic regression and multi-SAD regression models, respectively. ^b^For week *j*, $\rho \left( {\hat{u}_{.j} ,\hat{u}_{.j}^{*} } \right)$ where $\hat{u}_{RFI,ij}$ and $\hat{u}_{RFI,ij}^{*}$ correspond to the estimated breeding values for RFI for animal $i$ at week $j$ obtained with the phenotypic regression and multi-SAD regression models, respectively. Correlations calculated for 1229 phenotyped animals for the low RFI line and 1122 phenotyped animals for the high RFI line. Shaded area = 95% confidence interval

Figure 3 shows the estimates of the phenotypic and genetic correlations between RFI obtained with the phenotypic regression and multi-SAD regression models for the phenotyped animals by line and by week. Estimates of the phenotypic correlations were high and similar between the two lines, ranging from 0.87 to 0.98; they decreased from week 1 to 3 and then showed an inverted U-shaped profile, with a maximum value in week 7. The correlations between weekly EBV decreased over time, ranging from 0.96 to 0.79 in the High RFI line and from 0.95 to 0.77 in the Low RFI line.

**Fig. 4:**
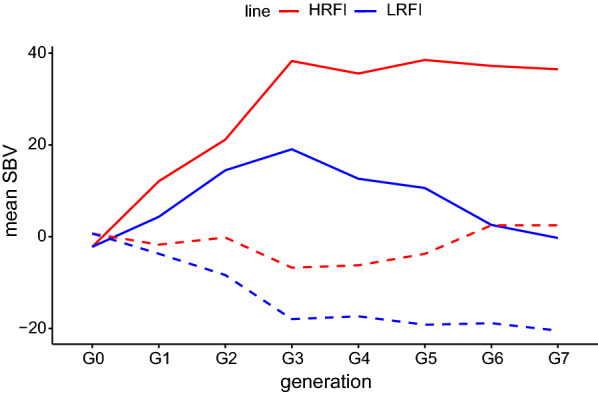
Changes in SBV1 (solid lines) and SBV2 (dashed lines) obtained with the multi-SAD regression model over generations of selection for the High and Low RFI lines

The trajectories of individual EBV over weeks were classified into three groups by a non-hierarchical k-mean analysis. These three patterns (see Additional file 5: Figure S3) differed by their means and slopes, which varied in the same direction, i.e. a higher mean was associated with a higher slope, and reciprocally. A Cohen’s kappa clustering agreement of 0.80 was found between the two models. The first two estimated breeding values SBV1 and SBV2 summarized the trajectory pattern into just two parameters: to some extent, SBV1 corresponded to the average RFI over the test period and SBV2 to the slope of the curve over time for each individual. Correlations between the first two SBV obtained with the two models are in Table 4. They were higher for SBV1 than for SBV2, similar in the two lines for SBV1 [0.83 (High RFI) and 0.82 (Low RFI)], and lower in the Low RFI line for SBV2 (0.76 versus 0.66). Figure 4 shows how SBV1 and SBV2 that were obtained with the multi-SAD model changed with selection. After seven generations of selection, animals of the Low RFI line had, on average, lower SBV1 and SBV2 than animals of the High RFI line, i.e. the trajectory of EBV over weeks for the Low line was characterised by a lower mean and a lower slope.

**Table 4:** Spearman correlations [0.95 confidence interval] per line between summarised estimated breeding values for RFI obtained with the phenotypic regression and the multi-SAD regression models

Summarised breeding value	High RFI line	Low RFI line
SBV1	0.83 [0.81, 0.85]	0.82 [0.79, 0.83]
SBV2	0.76 [0.73, 0.78]	0.66 [0.62, 0.69]

## Discussion

We chose the SAD approach to account for the genetic and environmental covariances between longitudinal production traits and FI in order to compute longitudinal RFI independent of production traits at the genetic level using the multi-SAD regression model, and to account for covariances between successive measurements in the phenotypic regression and multi-SAD regression models. Thus, applying the multi-SAD approach to components of RFI, we were able to obtain estimates of the covariance between FI and production traits over time and EBV for RFI independent of production traits. These properties are expected to be useful for selection [9] and for the study of patterns of feed efficiency over time at the genetic and phenotypic levels [34]. A random regression (RR) approach [19] could also have been used to account for these different covariances, as analysis of the longitudinal feed conversion ratio by phenotypic regression with RR or SAD approaches have recently been shown to produce similar results [15]. The RR approach has also been used to model RFI when FI is recorded longitudinally but only single records are available for production traits [35]. However, applying the RR approach to multiple traits requires estimation of more parameters than with the SAD approach and can lead to convergence problems [27]. The SAD approach has also other advantages over the RR approach, including providing a better fit to the data in many situations [17, 18, 24, 25], suffering less from the drawbacks reported for the RR model such as border effects [36], and from the challenge to properly estimate correlations that decrease over time [18]. In spite of these advantages compared to RR, we had to apply several constraints to the multi-SAD regression model to facilitate convergence: (1) we did not account for covariances between production traits (i.e. set to 0), and (2) we fixed the antedependence and innovation variance parameters for the production traits to their values estimated in the first steps of the analysis using single trait SAD models. It should also be noted that the units of measure for production traits and FI were chosen such that the variances of the traits were of similar magnitude to facilitate convergence, which is why ADG was expressed in 10× g/d, BF in 0.1× mm, etc. Initial analyses with fewer traits and time points showed that these constraints had no impact on the estimates of the genetic and environmental parameters for RFI.

It must be acknowledged that the multi-SAD regression model is a complex model that may have practical identifiability issues. Exploring the information matrix ${\mathbf{I}}\left( {\hat{\boldsymbol{{\upomega} }}} \right)$ helps to detect such problems for the unknown parameter vector (${{\boldsymbol{\upomega}}}$) [37]. The condition number (square root of the ratio of the first to the last eigenvalue) of the information matrix ${\mathbf{I}}\left( {\hat{\boldsymbol{{\upomega }}}} \right)$ of the SAD model used in our study was low, i.e. 26, which suggests that there was no practical identifiability issue for the data analyzed.

Using results from the multi-SAD model, we estimated RFI that is independent of production traits at the genetic level by regressing FI on production traits using genetic regression coefficients. Different RFI traits can be estimated from the results of the multi-SAD model. On the one hand, estimates of phenotypic regression coefficients corrected for fixed effects (i.e. extracted from the estimated phenotypic covariance matrix [9]) can be used to obtain a measure of RFI that is phenotypically but not genetically independent of production traits. On the other hand, estimates of the genetic regression coefficients can be used for the genetic part and estimates of the environmental regression coefficients for the environmental part to obtain a measure of RFI that is independent of production traits at both the genetic and phenotypic levels (see Additional file 1). However, the meaning and advantage of this measure of phenotypic RFI remain to be investigated. Here, we also derived RFI using a phenotypic regression model that does not account for the systematic effects that affect production traits. We investigated this model in spite of the known poor properties of the resulting EBV for RFI, i.e. the EBV are not independent of production traits, and the low accuracy of RFI parameter estimates [7, 38], because phenotypic regression of FI on ADG, MBW, and BF over the test period (i.e. one observation per animal) was used in the divergent selection experiment. In addition, it is the easiest and most common measure of RFI used in pigs and other species (e.g. cattle, sheep, chicken and even fish [39–42]), although some authors recommend to compute RFI from the genetic covariance matrix, such as Mebratie et al.[8]. Our results obtained with the multi-SAD and the phenotypic regression models were different. Given the known theoretical benefit of the multi-SAD model over the phenotypic regression model, our comparison suggests that applying the multi-SAD model would be preferable in practice. However, this must be confirmed by a more in-depth analysis of the costs (computing time) and benefits (improving RFI by genetic selection without detrimental effects on production traits at different time points) of applying the multi-SAD model over the phenotypic regression model. This will require evaluation of direct and correlated responses to selection [9] in their different dimensions (over time and generations) under scenarios that differ in the model and selection criteria used (subset of the SBV of interest, BV at a given time point, etc.).

Estimates of heritability of FI were moderate and tended to increase with age (see Additional file 3: Figure S1), in agreement with previous studies [7, 43, 44]. Estimates of heritability of the production traits were similar to previous estimates over the test period (i.e. one observation per animal) in the same population [45] and were within the range of estimates reported for three French pig breeds [46], for an Irish commercial cross [9], and as reviewed by Clutter [47]. Longitudinal estimates for these traits are more rarely reported in pigs. For FI and RFI over 10 consecutive weeks of growth in three pig breeds, Shirali et al. [36] reported estimates of heritability ranging from 0.13 to 0.23 and from 0.02 to 0.20, respectively. As in our study, heritability estimates tended to increase with time for FI but their estimates followed a U-shaped curve for RFI and were lower than in our study. Using random regression approaches with different splines and Legendre polynomials, Cai et al. [48] reported estimates of heritability for longitudinal FI, BW, BF between 90 and 210 days of age on Yorkshire RFI selection lines at Iowa State University that, in some cases, increased at the extreme ages, which is typically due to the limits of the RR model listed earlier in the Discussion. Their estimates, were higher than our estimates for BW and BF and slightly lower for FI.

Estimates of heritability for weekly RFI obtained with the phenotypic and multi-SAD regression models (Fig. 1) were within the range of heritabilities, from 0.13 to 0.38 [6, 7, 22, 46], that have been reported in the literature for average RFI in pigs over the test period. Heritability estimates obtained with the multi-SAD regression model tended to be lower (but only significantly lower in weeks 1, 2, 3, and 10) than those obtained with the phenotypic regression model. The lower estimates were the result of lower estimates of genetic variance, while estimates of environmental variance for RFI were similar for the two models. Concomitantly, in spite of a similar pattern, estimates of the genetic correlation between RFI at different time points obtained with the multi-SAD regression model were lower than those obtained with the phenotypic regression model. These differences (heritabilities and correlations) between these two models are probably the result of the genetic correlations with production traits that persist in the phenotypic regression model, whereas they were null in the multi-SAD regression model. Indeed, as demonstrated by Kennedy et al. [5], genetic independence between production traits and RFI obtained by phenotypic regression is achieved only when $\left( {1 - h_{P}^{2} } \right)cov\left( {\mathbf{u}_{FI} ,\mathbf{u}_{P} } \right) = h_{P}^{2} cov\left( {\mathbf{e}_{FI} ,\mathbf{e}_{P} } \right)$, where $P$ is the production trait. In our study, this condition was probably not met at all time points and, as expected, correlations of weekly EBV for RFI with weekly EBV for production traits tended to be weaker with the multi-SAD regression model than with the phenotypic regression model (see Additional file 6: Figure S4). The non-zero genetic correlations with production traits obtained with the phenotypic regression model may also explain the decreasing correlations between weekly EBV for RFI obtained with the two models over time (model differences that accumulate over time). Estimates of phenotypic correlations between weekly RFI obtained with the two models were weaker when phenotypic correlations of RFI with production traits were non-zero in the multi-SAD model (negative correlations with MBW at the beginning of the test period and slightly positive correlations with ADG at the end of the test period (see Additional file 4: Figure S2c), while RFI was phenotypically independent of the production traits with the phenotypic regression model. It should be noted that use of the phenotypic regression model at the test-period level (one single observation per animal) resulted in near genetic independence between RFI and production traits, with estimates of genetic correlations equal to − 0.05, 0.07 and 0.36 for RFI with ADG, BF, and MBW, respectively. In this case, the correlation between EBV obtained with the phenotypic regression and the multi-SAD regression model (adapted to a single observation for each trait) was high (0.96).

Estimates of the phenotypic regression coefficients obtained with the phenotypic regression model changed slightly over time (Table 3). For BF and MBW, the difference between the maximum and minimum phenotypic regression coefficients was not significantly different from 0 given the standard errors of the estimates, whereas for ADG, the regression coefficient estimates were significantly higher in weeks 6, 7, and 8 than in weeks 1, 2, and 3. However, when applying a linear regression model on these regression coefficients, none of the slopes were significantly different from zero (estimated slopes of 0.028, − 0.014, and − 0.004 for ADG, MWB, and BF, respectively, p_values > 0.30). This outcome is explained as follows: in the selection experiment, pigs were fed the same diet throughout the test period and the diet was formulated to cover the animals’ energy and amino acid requirements, such that feed intake was essentially driven by the net energy content of the feed [49]. Thus, the phenotypic relationships that we observed for FI with ADG, MBW, and BF were driven mainly by the energy costs of these traits. In most models, the energy costs of maintenance (MBW) and of fat and protein deposition are considered to be constant for an animal during growth [50], although in some cases individual factors have been shown to affect maintenance requirements (visceral mass versus lean tissue, for instance) [51]. In the final multi-SAD regression model, the cross-antedependence parameter functions (regression coefficients) were of degree 1 because higher degree cross-antedependence functions led to convergence problems or to spurious variance estimates. Thus, in the SAD model, genetic and environmental regression coefficients were considered to evolve linearly over time. Interpretation of changes in estimates of regression coefficients over time is more difficult for genetic and environmental regression coefficients than for phenotypic regression coefficients, and no information on these aspects is available in the literature. In practice, different factors could influence the partitioning between genetic and environmental components of the phenotypic covariation of FI with production traits over time. For example, competition for access to the feeder may increase over time for pigs raised in groups, which means that the importance of the environmental factors may increase relative to the genetic determinism of how feed is used for growth. In addition, estimates of regression coefficients varied most from the beginning to the end of the period for MBW, which was the trait with the smallest genetic and environmental variance estimates (ranging from 5 to 66 and from 17 to 45, respectively), while they varied least for BF, which was the trait with the largest genetic and environmental variance estimates (ranging from 18 to 203 and from 48 to 218, respectively). Thus, these changes in coefficients might have limited impacts on covariances of the trait with FI and on the resulting RFI.

For the purpose of comparison, missing phenotypes for production traits were replaced by their linear interpolations for both models. As stated in the Methods section, the multi-SAD regression model can cope with a substantial number of missing values for the production traits, which is another advantage of this model. In the phenotypic regression model, if one of the production traits is missing at a given time point for an animal, RFI is also missing, which would be problematic if many phenotypes were missing for the different production traits. In our case, applying the phenotypic regression model to the original dataset (i.e. the set with numerous missing phenotypes for production traits) led to spurious variance estimates [estimates of heritability of RFI were very high, up to 0.78, (see Additional file 7 Figure S5)]. Results of the multi-SAD regression model applied to the original dataset (the same multi-SAD regression model) are provided in Additional file 7: Figure S5 and Additional file 8: Figure S6. The heritability estimates that were obtained with the multi-SAD regression model applied to the initial dataset did not differ from those obtained with the dataset using linear interpolation of missing phenotypes. Patterns of estimates of the genetic and environmental regression coefficients over time were also similar between the two datasets, as were the slope estimates, although we did note a tendency for steeper slopes when phenotypes were missing. The main differences between the two datasets were evident for the SBV. When phenotypes were missing, the correlations between SBV obtained with the phenotypic regression and the multi-SAD regression model were weaker than the correlations obtained when models were applied to data with no missing phenotypes: 0.76 [0.73, 0.79], 0.80 [0.78, 0.82] for SBV1, and 0.48 [0.44, 0.52], 0.63 [0.60, 0.67] for SBV2 for the High and Low lines, respectively. This result indicates that, in practice, when applying the multi-SAD regression model to data with missing records, selection based on SBV will differ markedly from selection on estimates obtained with the phenotypic regression model, particularly regarding the profile of RFI changes over time (SBV2). Values of the first eigenvector with the multi-SAD model, which was used to compute SBV1, were all positive and increased with time, while the sign of the values of the second eigenvector changed at week 7. This means that selection for SBV1 would lead to selection in the same direction at all time points, while selection for SBV2 would have opposite effects for the beginning versus the end of the test period. In our study, animals were divergently selected based on RFI obtained by phenotypic regression over the test period (one record per animal). Changes in SBV1 and SBV2 obtained with the multi-SAD model showed that this selection had, as expected, an impact on the average level of RFI over the test period, but also on the dynamics of RFI during the test period. This result is confirmed by the distribution of the individuals from the two lines into three trajectory patterns: 90% of the animals that were classified into the cluster with the lowest mean and slope were from the Low RFI line, while 94% of the animals that were classified into the cluster with the highest mean and slope were from the High RFI line. A previous study in the same population on the impact of selection for RFI at the test period level on the trajectory of feed conversion ratio reported similar results [15]. In practice, selection for RFI based on SBV1 and SBV2 allows control of the average level of RFI, as well as its changes with age. Nonetheless, further research is needed to identify the desired pattern of the RFI trajectory to select for. Indeed, is it preferable to select animals with a constant RFI over time, or an RFI that increases or decreases with age? To answer this question, studies on the covariation of longitudinal RFI with other traits of interest, such as carcass composition and meat quality, are needed, as well as on the evaluation of the economic impact of RFI at different ages.

## Conclusions

The multi-SAD regression model is preferred over the phenotypic regression model for analysis of longitudinal RFI because the multi-SAD regression model can be applied even when phenotypes for production traits are missing. In addition, RFI obtained with the multi-SAD regression model are genetically independent of production traits at all time points, in contrast to the phenotypic regression model. Selection on SBV for RFI does not result in selection of the same animals when based on these two models.

## Supplementary Information

**Additional file 1.** Demonstration of the calculation of RFI with the multi-SAD regression model.

**Additional file 2.** SAD functions retained for the phenotypic regression and multi-SAD regression models.

**Additional file 3: Figure S1.** Heritability over weeks of feed intake (red line), average daily gain (orange line), metabolic body weight (blue line) and backfat thickness (green line) obtained with the multi-SAD model.

**Additional file 4: Figure S2.** Genetic (a) and phenotypic (b) correlations for feed intake and production traits and phenotypic correlation for RFI and production traits (c) obtained with the multi-SAD model.

**Additional file 5: Figure S3.** Individual EBV trajectories and group trajectories resulting from non-hierarchical k-mean clustering analysis with 3 clusters obtained with the phenotypic and the multi-SAD regression models.

**Additional file 6: Figure S4.** Weekly correlations between estimated breeding values of residual feed intake and production traits (average daily gain in blue, metabolic body weight in red, backfat in green) obtained for phenotyped animals with the phenotypic regression (solid lines) and multi-SAD regression (dashed lines) models.

**Additional file 7: Figure S5.** Heritabilities obtained with the phenotypic and multi SAD regression model applied to data with some missing weekly production traits (red line = phenotypic regression, blue line = multi SAD) and with linear interpolation of missing production traits (green line = phenotypic regression, black line = multi-SAD).

**Additional file 8: Figure S6.** Genetic (solid lines) and environmental (dashed lines) regression coefficient estimates for average daily gain (in blue), metabolic body weight (in orange), and backfat (in green) obtained with the multi-SAD regression model applied to data with linear interpolation of missing production trait phenotypes (darker shade of each colour) and with (lighter shade of each colour) some missing weekly production trait phenotypes.

## Data Availability

The datasets used and analysed in the current study are available upon reasonable request to Hélène Gilbert (helene.gilbert@inrae.fr). The program used to run the SAD model is freely available on the Zenodo platform (https://zenodo.org/record/896377).
