# Supplementary material for: New residual feed intake criterion for longitudinal data
Source: Genet Sel Evol. 2021 Jun 25;53:53. doi: 10.1186/s12711-021-00641-2 (PMC8235855; doi:10.1186/s12711-021-00641-2)
Supplement: Supplementary file 1 — Additional file 1. Demonstration of the calculation of RFI with the multi-SAD regression model. [file 12711_2021_641_MOESM1_ESM.pdf]

Additional file 1 : Demonstration of the calculation of RFI*

The RFI* is obtained by correcting FI by ADG, MBW and BF at each time points using genetic regression coefficients:

For the genetic component of RFI*,

at time t_1, $u_{FI,i1} = b_{u,ADG1}u_{ADG,i1} + b_{u,MBW1}u_{MBW,i1} + b_{u,BF1}u_{BF,i1} + \varepsilon_{uFI,i1}$

thus $u_{RFI*,i1} = \varepsilon_{uFI,i1}$

At time t_2,

$$u_{FI,i2} = \theta_{uFI,2}u_{FI,i1} + b_{u,ADG2}u_{ADG,i2} + b_{u,MBW2}u_{MBW,i2} + b_{u,BF2}u_{ABFi2} + \varepsilon_{uFI,i2}$$

$$\Leftrightarrow u_{FI,i2} = \theta_{uFI,2}\left(b_{u,ADG1}u_{ADG,i1} + b_{u,MBW1}u_{MBW,i1} + b_{u,BF1}u_{BF,i1} + \varepsilon_{uFI,i1}\right) + b_{u,ADG2}u_{ADG,i2} + b_{u,MBW2}u_{MBW,i2} + b_{u,BF2}u_{ABFi2} + \varepsilon_{uFI,i2}$$

Computing RFI* by correcting FI by all components related to ADG,MBW and BF, gives:

$$u_{RFI*,i2} = u_{FI,i2} - \theta_{uFI,2}\left(b_{u,ADG1}u_{ADG,i1} + b_{u,MBW1}u_{MBW,i1} + b_{u,BF1}u_{BF,i1}\right) - \left(b_{u,ADG2}u_{ADG,i2} + b_{u,MBW2}u_{MBW,i2} + b_{u,BF2}u_{ABFi2}\right)$$

$$\Leftrightarrow u_{RFI*,i2} = \theta_{uFI,2}\varepsilon_{uFI,i1} + \varepsilon_{uFI,i2}$$

This equation is useful to compute the genetic variance of RFI* as a function of the innovation variances and antedependence parameters. However, since the error terms $\varepsilon_{uFI,ij}$ are not predicted by the program, to be able to predict the genetic value of RFI*, $u_{RFI,i2}$ has to be expressed as a function of the other genetic effects. Replacing $\varepsilon_{uFI,i1}$ by $u_{RFI,i1}$ and $\varepsilon_{uFI,i2}$ by $u_{FI,i2} - \theta_{uFI,2}u_{FI,i1} - b_{u,ADG2}u_{ADG,i2} - b_{u,MBW2}u_{MBW,i2} - b_{u,BF2}u_{BFi2}$ in the preceding equation, gives:

$$u_{RFI*,i2} = \theta_{uFI,2}u_{RFI*,i1} + u_{FI,i2} - \theta_{uFI,2}u_{FI,i1} - b_{u,ADG2}u_{ADG,i2} - b_{u,MBW2}u_{MBW,i2} - b_{u,BF2}u_{BFi2}$$

$$\Leftrightarrow u_{RFI*,i2} = u_{FI,i2} + \theta_{uFI,2}\left(u_{RFI*,i1} - u_{FI,i1}\right) - b_{u,ADG2}u_{ADG,i2} - b_{u,MBW2}u_{MBW,i2} - b_{u,BF2}u_{BFi2}$$

The same reasoning was used for $u_{RFI*,i3}$:

$$u_{FI,i3} = \theta_{uFI,3}u_{FI,i2} + b_{u,ADG3}u_{ADG,i3} + b_{u,MBW3}u_{MBW,i3} + b_{u,BF3}u_{BF,i3} + \varepsilon_{uFI,i3}$$

$$\Leftrightarrow u_{FI,i3} = \theta_{uFI,3}\theta_{uFI,2}\left(b_{u,ADG1}u_{ADG,i1} + b_{u,MBW1}u_{MBW,i1} + b_{u,BF1}u_{BF,i1}\right) + \theta_{uFI,3}\theta_{uFI,2}\varepsilon_{uFI,i1} + \theta_{uFI,3}\left(b_{u,ADG2}u_{ADG,i2} + b_{u,MBW2}u_{MBW,i2} + b_{u,BF2}u_{BF,i2}\right) + \theta_{uFI,3}\varepsilon_{uFI,i2} + b_{u,ADG3}u_{ADG,i3} + b_{u,MBW3}u_{MBW,i3} + b_{u,BF3}u_{BF,i3} + \varepsilon_{uFI,i3}$$

Thus

$$u_{RFI*,i3} = u_{FI,i3} - \left[\theta_{uFI,3}\theta_{uFI,2}\left(b_{u,ADG1}u_{ADG,i1} + b_{u,MBW1}u_{MBW,i1} + b_{u,BF1}u_{BF,i1}\right) + \theta_{uFI,3}\left(b_{u,ADG2}u_{ADG,i2} + b_{u,MBW2}u_{MBW,i2} + b_{u,BF2}u_{BF,i2}\right) + b_{u,ADG3}u_{ADG,i3} + b_{u,MBW3}u_{MBW,i3} + b_{u,BF3}u_{BF,i3}\right]$$

$$\Leftrightarrow u_{RFI*,i3} = \theta_{uFI,3}\theta_{uFI,2}\varepsilon_{uFI,i1} + \theta_{uFI,3}\varepsilon_{uFI,i2} + \varepsilon_{uFI,i3}$$

Since $\theta_{uFI,2}\varepsilon_{uFI,i1} + \varepsilon_{uFI,i2} = u_{RFI*,i2}$ and $\varepsilon_{uFI,i3} = u_{FI,i3} - \theta_{uFI,3}u_{FI,i2} - b_{u,ADG3}u_{ADG,i3} - b_{u,MBW3}u_{MBW,i3} - b_{u,BF3}u_{BF,i3}$, we obtain:

$$u_{RFI*,i3} = u_{FI,i3} - \theta_{uFI,3}\left(u_{RFI*,i2} - u_{FI,i2}\right) - b_{u,ADG3}u_{ADG,i3} - b_{u,MBW3}u_{MBW,i3} - b_{u,BF3}u_{BF,i3}$$

Recursively, we define the general formula for predicting the genetic part of RFI* at time $t_j > t_1$ as

$$u_{RFI*,ij} = u_{FI,ij} - \theta_{uFI,j}\left(u_{RFI*,i(j-1)} - u_{FI,i(j-1)}\right) - b_{u,ADGj}u_{ADG,ij} - b_{u,MBWj}u_{MBW,ij} - b_{u,BFj}u_{BF,ij}$$

And the general formula for its variance as:

$$\sigma^2_{uRFI*,j} = \sum_{k=1}^{j-1} \prod_{l=k+1}^{j} \theta_{uFI,l}\, \sigma^2_{\varepsilon uFI,k} + \sigma^2_{\varepsilon uFI,j}$$

It should be noted that the general formula for the genetic variance of RFI* is the same as the one of a single trait SAD model applied to FI (ignoring the cross-antedependence with the production parameters). The inverse of the genetic covariance matrix of RFI* can then be easily obtained by the same Cholesky decomposition as for single trait analysis.

For the environmental part of RFI*, genetic coefficients of regression are also used, leading to:

$e_{RFI*,i1} = e_{FI,i1} - b_{u,ADG1}e_{ADG,i1} - b_{u,MBW1}e_{MBW,i1} - b_{e,BF1}u_{BF,i1}$, at time t_1.

At time t_2,

$$e_{RFI*,i2} = e_{FI,i2} - b_{u,ADG2}e_{ADG,i2} - b_{u,MBW2}e_{MBW,i2} - b_{e,BF2}u_{BF,i2} - \theta_{eFI,2}b_{u,ADG1}e_{ADG,i1} - \theta_{eFI,2}b_{u,MBW1}e_{MBW,i1} - \theta_{eFI,2}b_{e,BF1}u_{BF,i1}$$

$$\Leftrightarrow e_{RFI*,i2} = \theta_{eFI,2}e_{FI,i1} + b_{e,ADG2}e_{ADG,i2} + b_{e,MBW2}e_{MBW,i2} + b_{e,BF2}u_{BF,i2} + \varepsilon_{eFI,i2} - b_{u,ADG2}e_{ADG,i2} - b_{u,MBW2}e_{MBW,i2} - b_{e,BF2}u_{BF,i2} - \theta_{eFI,2}b_{u,ADG1}e_{ADG,i1} - \theta_{eFI,2}b_{u,MBW1}e_{MBW,i1} - \theta_{eFI,2}b_{e,BF1}u_{BF,i1}$$

$$\Leftrightarrow e_{RFI*,i2} = \theta_{eFI,2}e_{RFI*,i1} + b_{e,ADG2}e_{ADG,i2} + b_{e,MBW2}e_{MBW,i2} + b_{e,BF2}u_{BF,i2} + \varepsilon_{eFI,i2} - b_{u,ADG2}e_{ADG,i2} - b_{u,MBW2}e_{MBW,i2} - b_{e,BF2}u_{BF,i2}$$

$$\Leftrightarrow e_{RFI*,i2} = e_{FI,i2} + \theta_{eFI,2}\left(e_{RFI*,i1} - e_{FI,i1}\right) - b_{u,ADG2}e_{ADG,i2} - b_{u,MBW2}e_{MBW,i2} - b_{e,BF2}u_{BF,i2}$$

Recursively, we define the general formula for predicting the environmental part of RFI* at time $t_j > t_1$ as

$$e_{RFI*,ij} = e_{FI,ij} - \theta_{eFI,j}\left(e_{RFI*,i(j-1)} - e_{FI,i(j-1)}\right) - b_{u,ADGj}e_{ADG,ij} - b_{u,MBWj}e_{MBW,ij} - b_{u,BFj}e_{BF,ij}$$

The covariance matrix of the environmental part of RFI* is obtained using the following covariance function: $\boldsymbol{BP_TB'}$, where $\boldsymbol{P_T}$ is the environmental covariance matrix for FI and production traits and $\boldsymbol{B}$ is a lower triangular matrix of regression coefficients: $\boldsymbol{B} = \begin{bmatrix} \boldsymbol{I_{ADG}} & & & \\ \boldsymbol{0} & \boldsymbol{I_{MBW}} & & \\ \boldsymbol{0} & \boldsymbol{0} & \boldsymbol{I_{BF}} & \\ \boldsymbol{b_{eADF/FI}} & \boldsymbol{b_{eMBW/FI}} & \boldsymbol{b_{eBF/FI}} & \boldsymbol{I_{FI}} \end{bmatrix}$, $\boldsymbol{b_{es/FI}}$ ($s \in \{ADG, MBW, BF\}$) are lower triangular matrices with the negative of the genetic cross-antedependence parameters (genetic regression coefficients) on the diagonal and the negatives of the product of environmental antedependence parameters with genetic cross-antedependence parameters as off-diagonal entries ($for\ cell\ (i,j), i < j{:} -\prod_{k=2}^{j}\theta_{eFI,k}b_{u,si}$):

$$\boldsymbol{b_{s/FI}} = \begin{bmatrix} -b_{u,s1} & & & & \\ -\theta_{eFI,2}b_{u,s1} & \cdots & & & \\ -\prod_{k=2}^{j}\theta_{eFI,k}b_{u,s1} & \cdots & -b_{u,sj} & & \\ \vdots & \vdots & \vdots & \cdots & \\ \cdots & \cdots & -\prod_{k=2}^{n}\theta_{eFI,k}b_{u,sj} & \cdots & -b_{u,sn} \end{bmatrix}$$ for n times of measurements

It should be noted that it is possible to compute environmental part of RFI independent from production traits at all time points using the same reasoning as for the genetic part of RFI* at time t_j leading to:

$$e_{RFI**,ij} = e_{FI,ij} - \theta_{eFI,j}\left(e_{RFI*,i(j-1)} - e_{FI,i(j-1)}\right) - b_{e,ADGj}e_{ADG,ij} - b_{e,MBWj}e_{MBW,ij} - b_{e,BFj}e_{BF,ij}$$

And

$$\sigma^2_{eRFI**,j} = \sum_{k=1}^{j-1}\prod_{l=k+1}^{j}\theta_{eFI,l}\,\sigma^2_{\varepsilon eFI,k} + \sigma^2_{\varepsilon eFI,j}$$

[1] David I., Garreau H., Balmisse E., Billon Y., Canario L., Multiple-trait structured antedependence model to study the relationship between litter size and birth weight in pigs and rabbits, Genetics Selection Evolution. 49 (2017) 11. 10.1186/s12711-017-0288-3.
