# Supplementary material for: New residual feed intake criterion for longitudinal data
Source: Genet Sel Evol. 2021 Jun 25;53:53. doi: 10.1186/s12711-021-00641-2 (PMC8235855; doi:10.1186/s12711-021-00641-2)
Supplement: Supplementary file 2 — Additional file 2. SAD functions retained for the phenotypic regression and multi-SAD regression models. [file 12711_2021_641_MOESM2_ESM.pdf]

Additional file 2 : SAD functions retained for the phenotypic regression and innovative regression models

Phenotypic regression model:

$$u_{RFI,ij} = (a_{uRFI,0} + a_{uRFI,1}t_j)u_{RFI,i(j-1)} + \varepsilon_{uRFI,ij}, \boldsymbol{\varepsilon}_{uRFI,.j} \sim N\left(0, \boldsymbol{A}exp(d_{uRFI,0} + d_{uRFI,1}t_j)\right)$$
$$e_{RFI,ij} = (a_{eRFI,0} + a_{eRFI,1}t_j)e_{RFI,i(j-1)} + \varepsilon_{eRFI,ij}, \boldsymbol{\varepsilon}_{uRFI,.j} \sim N\left(0, \boldsymbol{I}exp(d_{eRFI,0} + d_{eRFI,1}t_j + d_{eRFI,2}t_j^2)\right)$$

Innovative regression model:

$$u_{ADG,ij} = a_{uADG,0}u_{ADG,i(j-1)} + \varepsilon_{uADG,ij}$$
$$u_{MBW,ij} = a_{uMBW,0}u_{MBW,i(j-1)} + \varepsilon_{uMBW,ij}$$
$$u_{BF,ij} = a_{uBF,0}u_{BF,i(j-1)} + \varepsilon_{uBF,ij}$$
$$u_{FI,ij} = (a_{uFI,0} + a_{uFI,1}t_j)u_{FI,i(j-1)} + (c_{uADG,0} + c_{uADG,1}t_j)u_{ADG,ij} + (c_{uMBW,0} + c_{uMBW,1}t_j)u_{MBW,ij} + (c_{uBF,0} + c_{uBF,1}t_j)u_{BF,ij} + \varepsilon_{uFI,ij}$$

$$\boldsymbol{\varepsilon}_{uADG,.j} \sim N\left(0, \boldsymbol{A}exp(d_{uADG,0})\right)$$
$$\boldsymbol{\varepsilon}_{uMBW,.j} \sim N\left(0, \boldsymbol{A}exp(d_{uMBW,0})\right)$$
$$\boldsymbol{\varepsilon}_{uBF,.j} \sim N\left(0, \boldsymbol{A}exp(d_{uBF,0})\right)$$
$$\boldsymbol{\varepsilon}_{uFI,.j} \sim N\left(0, \boldsymbol{A}exp(d_{uFI,0} + d_{uFI,1}t_j)\right)$$

$$e_{ADG,ij} = a_{eADG,0}e_{ADG,i(j-1)} + \varepsilon_{eADG,ij}$$
$$e_{MBW,ij} = a_{eMBW,0}e_{MBW,i(j-1)} + \varepsilon_{eMBW,ij}$$
$$e_{BF,ij} = a_{eBF,0}e_{BF,i(j-1)} + \varepsilon_{eBF,ij}$$
$$e_{FI,ij} = (a_{eFI,0} + a_{eFI,1}t_j)e_{FI,i(j-1)} + (c_{eADG,0} + c_{eADG,1}t_j)e_{ADG,ij} + (c_{eMBW,0} + c_{eMBW,1}t_j)e_{MBW,ij} + (c_{eBF,0} + c_{eBF,1}t_j)e_{BF,ij} + \varepsilon_{eFI,ij}$$

$$\boldsymbol{\varepsilon}_{eADG,.j} \sim N\left(0, \boldsymbol{I}exp(d_{eADG,0})\right)$$
$$\boldsymbol{\varepsilon}_{eMBW,.j} \sim N\left(0, \boldsymbol{I}exp(d_{eMBW,0} + d_{eMBW,1}t_j)\right)$$
$$\boldsymbol{\varepsilon}_{eBF,.j} \sim N\left(0, \boldsymbol{I}exp(d_{eBF,0} + d_{eBF,1}t_j)\right)$$
$$\boldsymbol{\varepsilon}_{uFI,.j} \sim N\left(0, \boldsymbol{I}exp(d_{eFI,0} + d_{eFI,1}t_j + +d_{eFI,1}t_j^2)\right)$$
