# Supplementary material for: New residual feed intake criterion for longitudinal data
Source: Genet Sel Evol. 2021 Jun 25;53:53. doi: 10.1186/s12711-021-00641-2 (PMC8235855; doi:10.1186/s12711-021-00641-2)

Additional file 3: Figure S1 Heritability over weeks of feed intake (red line), average daily gain (orange line), metabolic body weight (blue line) and backfat thickness (green line) obtained with the multi-SAD model

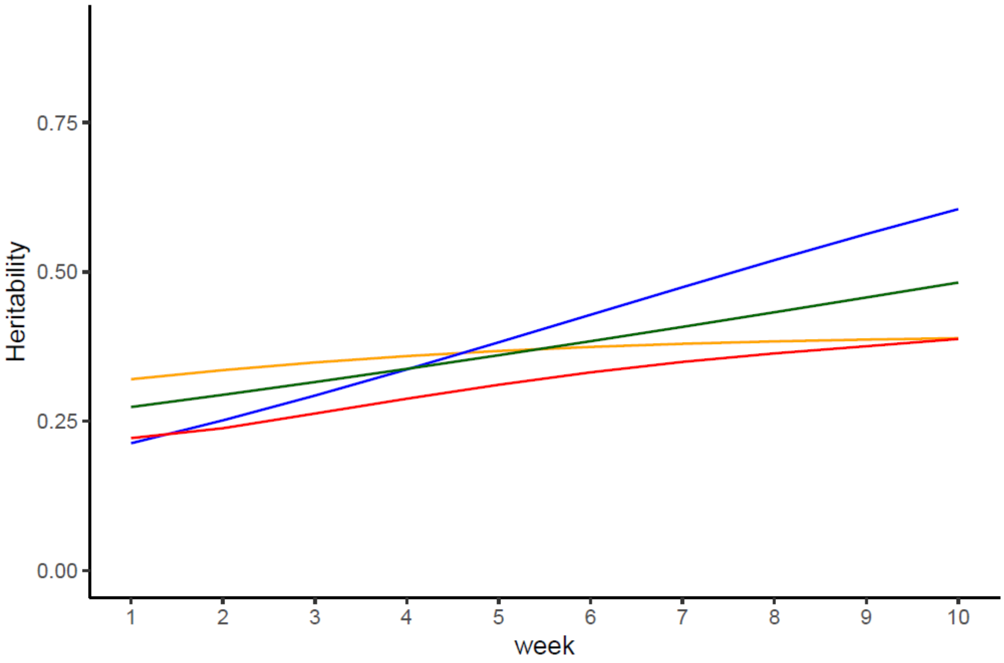

Supplement: Supplementary file 3 — Additional file 3: Figure S1. Heritability over weeks of feed intake (red line), average daily gain (orange line), metabolic body weight (blue line) and backfat thickness (green line) obtained with the multi-SAD model. [file 12711_2021_641_MOESM3_ESM.pdf]
