# Supplementary material for: New residual feed intake criterion for longitudinal data
Source: Genet Sel Evol. 2021 Jun 25;53:53. doi: 10.1186/s12711-021-00641-2 (PMC8235855; doi:10.1186/s12711-021-00641-2)

Additional file 4: Figure S2 genetic (a) and phenotypic (b) correlations for feed intake and production traits and phenoypic correlation for RFI and production traits (c) obtained with the multi-SAD model

(a)

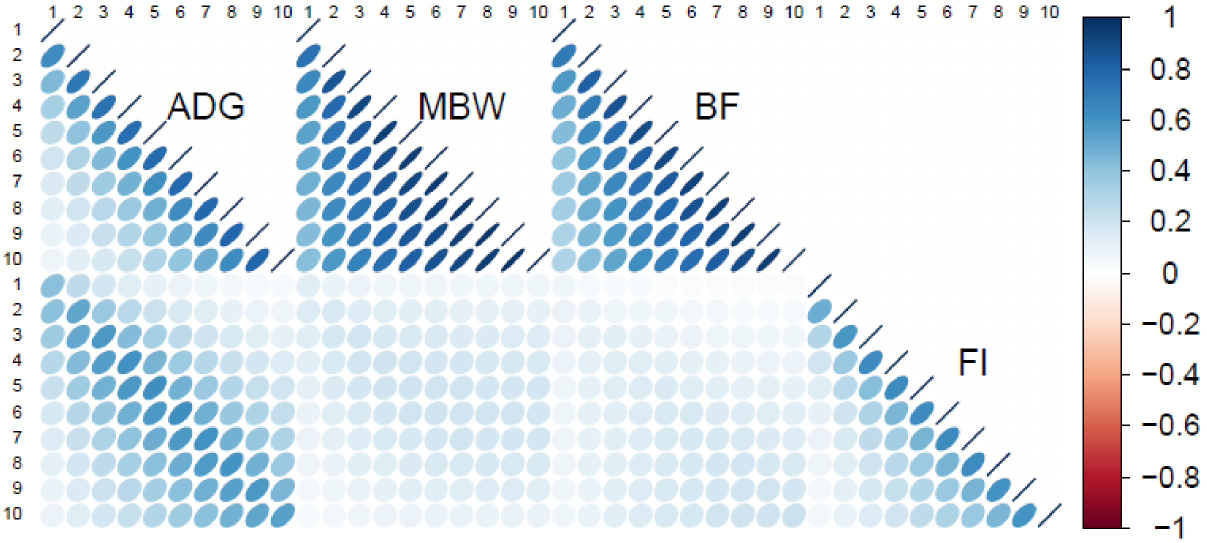

(b)

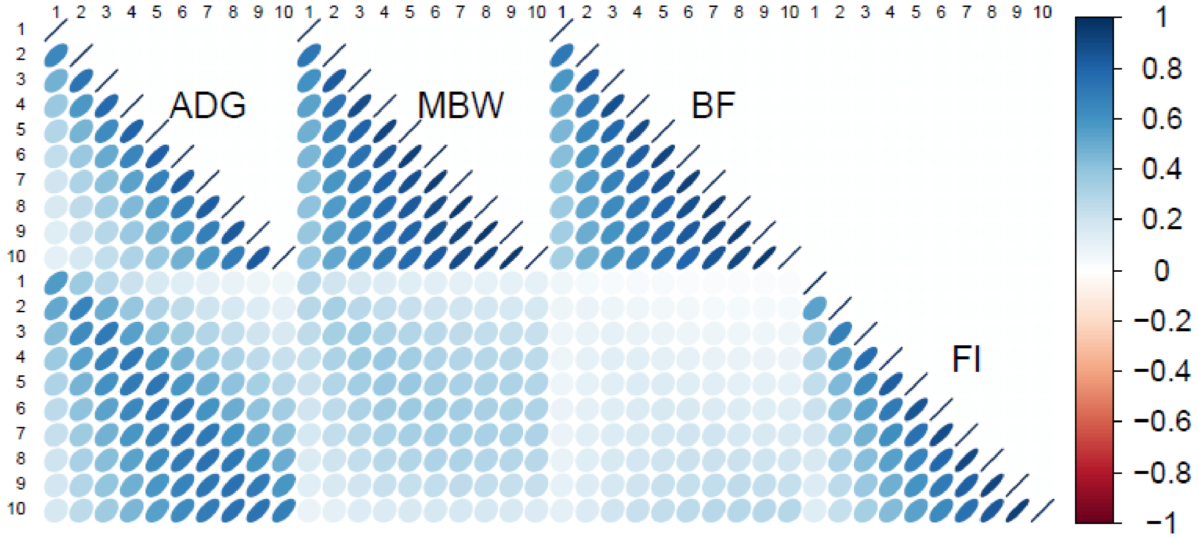

(c)

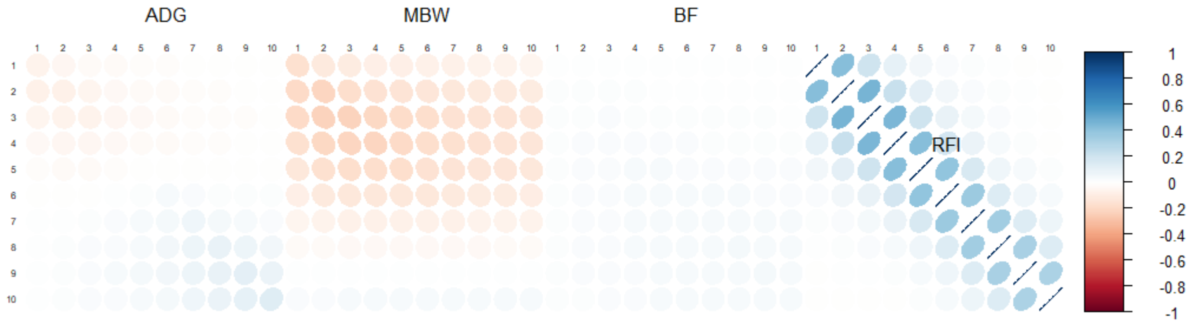

Supplement: Supplementary file 4 — Additional file 4: Figure S2. Genetic (a) and phenotypic (b) correlations for feed intake and production traits and phenotypic correlation for RFI and production traits (c) obtained with the multi-SAD model. [file 12711_2021_641_MOESM4_ESM.pdf]
