# Supplementary material for: New residual feed intake criterion for longitudinal data
Source: Genet Sel Evol. 2021 Jun 25;53:53. doi: 10.1186/s12711-021-00641-2 (PMC8235855; doi:10.1186/s12711-021-00641-2)

**Additional file 5: Figure S3: Individual EBV trajectories (black) and group trajectories resulting from nonhierarchical k-mean clustering analyses with 3 clusters obtained with the phenotypic regression (a) and multi-SAD regression models (b)**

(a)

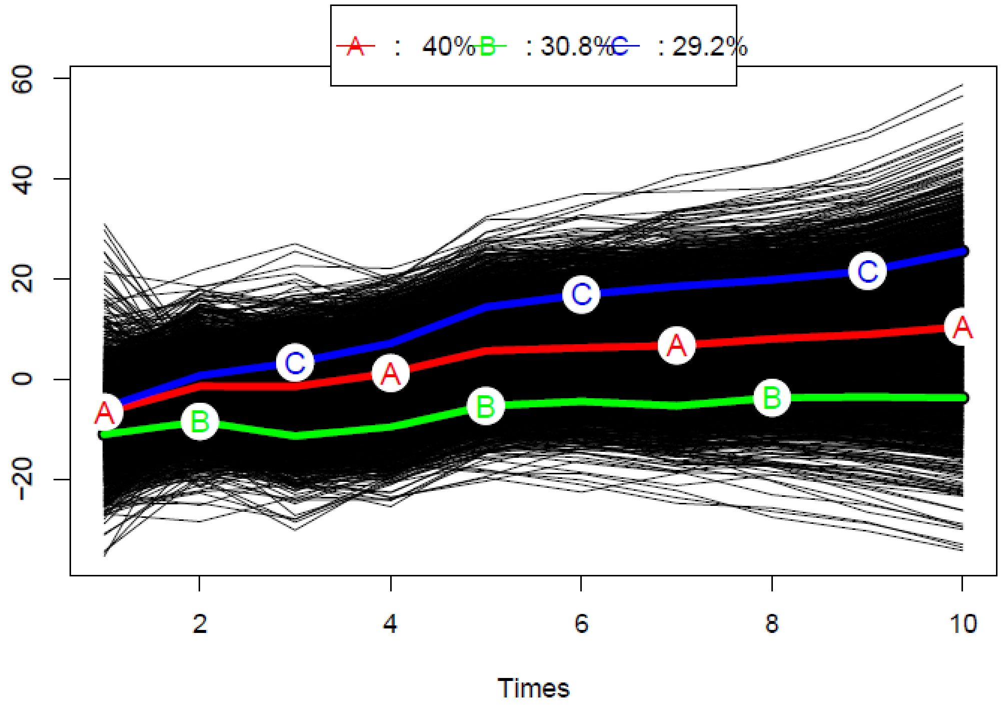


(b)

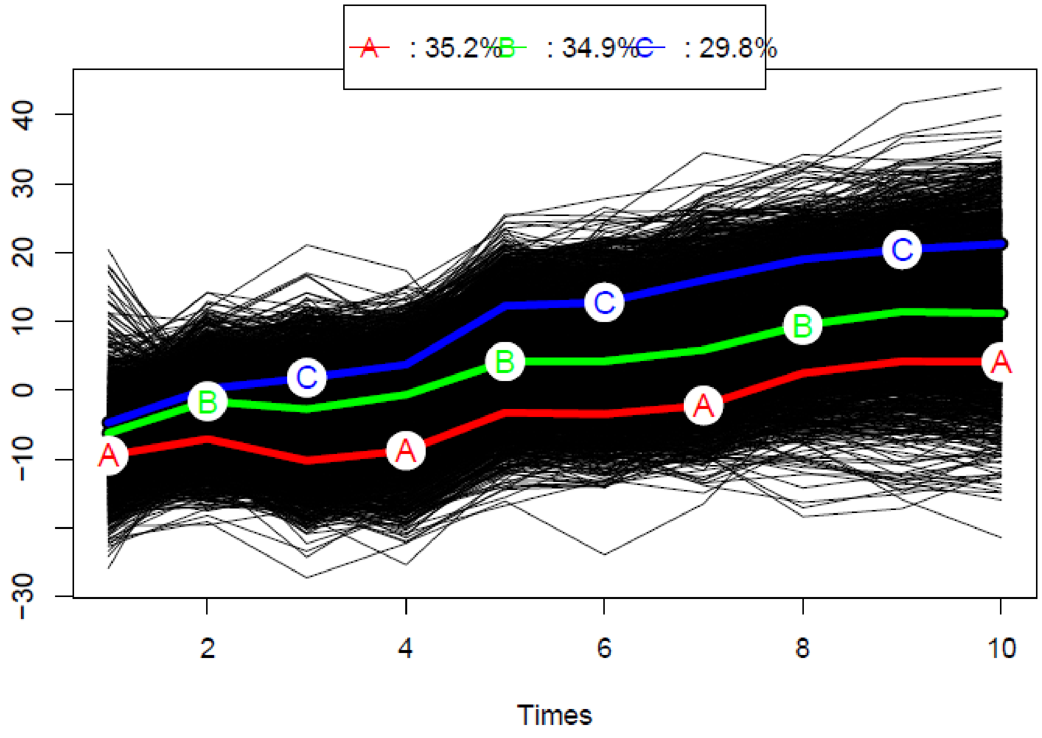

Supplement: Supplementary file 5 — Additional file 5: Figure S3. Individual EBV trajectories and group trajectories resulting from non-hierarchical k-mean clustering analysis with 3 clusters obtained with the phenotypic and the multi-SAD regression models. [file 12711_2021_641_MOESM5_ESM.pdf]
