# Supplementary material for: New residual feed intake criterion for longitudinal data
Source: Genet Sel Evol. 2021 Jun 25;53:53. doi: 10.1186/s12711-021-00641-2 (PMC8235855; doi:10.1186/s12711-021-00641-2)

Additional file 6: Figure S4 Weekly correlations between estimated breeding values of residual feed intake and production traits (average daily gain in blue, metabolic body weight in red, backfat in green) obtained for phenotyped animals with the phenotypic regression (solid lines) and multi-SAD regression (dashed lines) models

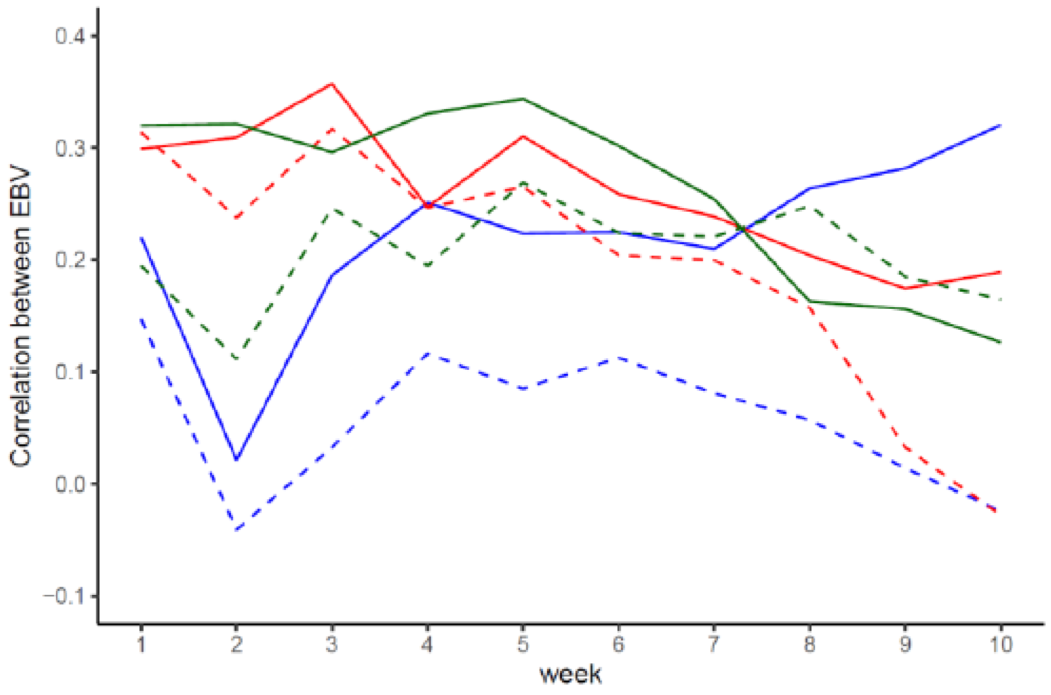

Supplement: Supplementary file 6 — Additional file 6: Figure S4. Weekly correlations between estimated breeding values of residual feed intake and production traits (average daily gain in blue, metabolic body weight in red, backfat in green) obtained for phenotyped animals with the phenotypic regression (solid lines) and multi-SAD regression (dashed lines) models. [file 12711_2021_641_MOESM6_ESM.pdf]
