# Supplementary material for: New residual feed intake criterion for longitudinal data
Source: Genet Sel Evol. 2021 Jun 25;53:53. doi: 10.1186/s12711-021-00641-2 (PMC8235855; doi:10.1186/s12711-021-00641-2)

Additional file 7: figure S5 Heritabilities obtained with the phenotypic and multi SAD regression model applied to data with some missing weekly production traits (red line=phenotypic regression, blue line=multi SAD) and with linear interpolation of missing production traits (green line=phenotypic regression, black line=multi-SAD).

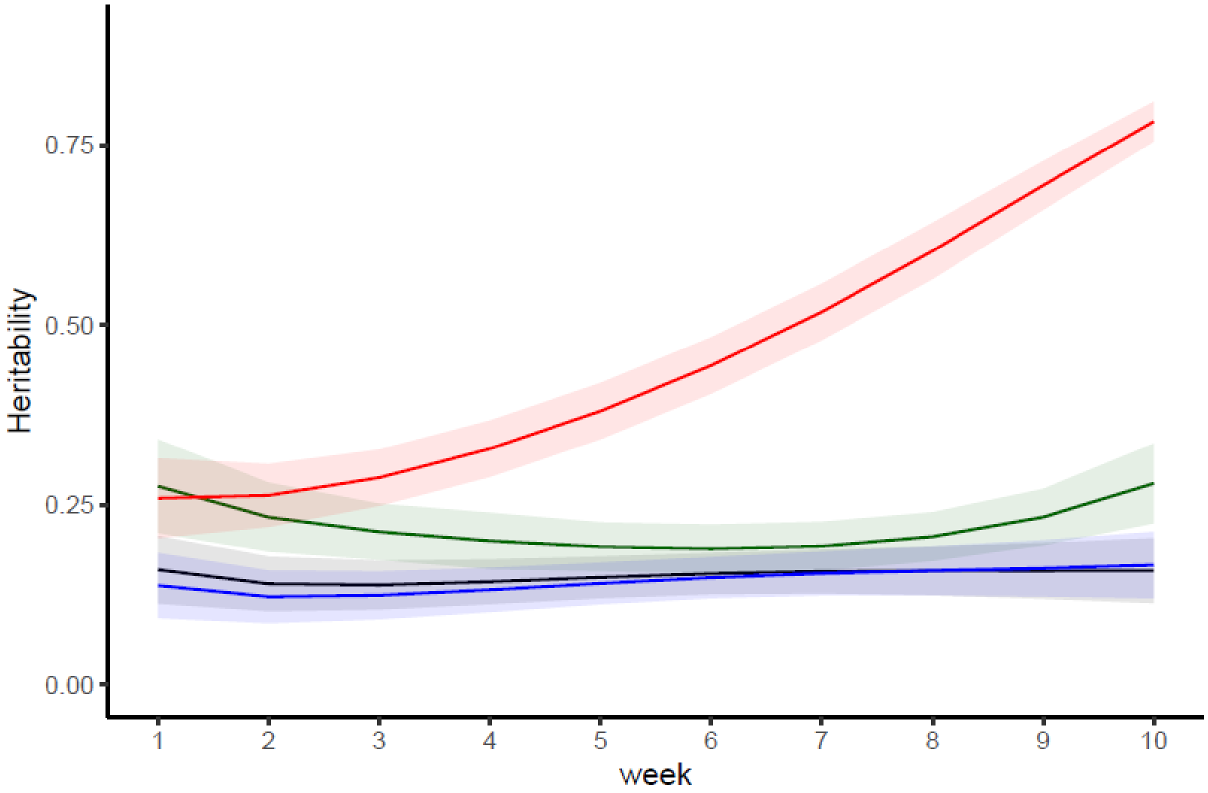

Supplement: Supplementary file 7 — Additional file 7: Figure S5. Heritabilities obtained with the phenotypic and multi SAD regression model applied to data with some missing weekly production traits (red line = phenotypic regression, blue line = multi SAD) and with linear interpolation of missing production traits (green line = phenotypic regression, black line = multi-SAD). [file 12711_2021_641_MOESM7_ESM.pdf]
