# Supplementary material for: New residual feed intake criterion for longitudinal data
Source: Genet Sel Evol. 2021 Jun 25;53:53. doi: 10.1186/s12711-021-00641-2 (PMC8235855; doi:10.1186/s12711-021-00641-2)

Additional file 8: Figure S6 Genetic (solid lines) and environmental (dashed lines) regression coefficient estimates for average daily gain (in blue), metabolic body weight (in orange), and backfat (in green) obtained with the multi-SAD regression model applied to data with linear interpolation of missing production trait phenotypes (darker shade of each colour) and with (lighter shade of each colour) some missing weekly production trait phenotypes.

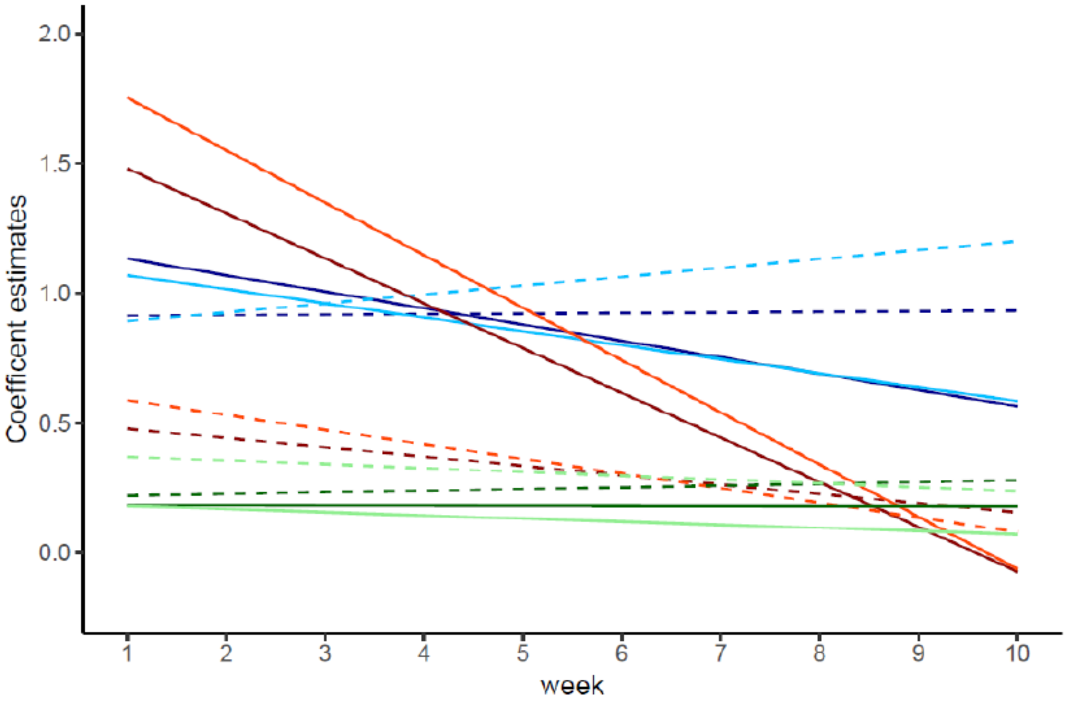

Supplement: Supplementary file 8 — Additional file 8: Figure S6. Genetic (solid lines) and environmental (dashed lines) regression coefficient estimates for average daily gain (in blue), metabolic body weight (in orange), and backfat (in green) obtained with the multi-SAD regression model applied to data with linear interpolation of missing production trait phenotypes (darker shade of each colour) and with (lighter shade of each colour) some missing weekly production trait phenotypes. [file 12711_2021_641_MOESM8_ESM.pdf]
